# Changing Trends in the Global Burden of Cataract Over the Past 30 Years: Retrospective Data Analysis of the Global Burden of Disease Study 2019

**DOI:** 10.2196/47349

**Published:** 2023-12-05

**Authors:** Bo Jiang, Tianhong Wu, Weiming Liu, Gaoqin Liu, Peirong Lu

**Affiliations:** 1 Department of Ophthalmology The First Affiliated Hospital of Soochow University Suzhou China

**Keywords:** burden, cataract, disability-adjusted life-years, human development index, prediction, risk factor

## Abstract

**Background:**

Cataracts now account for the largest proportion of the global burden of blindness and vision loss. Understanding the changing trends in the global burden of cataracts over the past 30 years and the next 15 years is of clear significance for the prevention and control of cataracts in key populations. As far as we know, research on the future burden of cataracts is lacking.

**Objective:**

This study aims to assess the global burden of cataracts over the past 30 years by using age-period-cohort modeling and to estimate trends in the next 15 years.

**Methods:**

Data were obtained from the Global Burden of Disease Study 2019, the United Nations Development Programme, and the WHO (World Health Organization) Global Health Observatory data repository. The assessment of trends and disparities in the number and rate of disability-adjusted life years (DALYs) for cataracts from 1990 to 2019 was conducted. The association between the age-standardized DALY rate (ASDR) and the socio-demographic index (SDI), human development index (HDI), national levels of particulate matter <2.5 μm in diameter (PM_2.5_), and ambient ultraviolet radiation (UVR) was determined using linear regression analysis. Additionally, we used the Nordpred (Harald Fekjær and Bjørn Møller) age-period-cohort model to predict the cataract burden from 2020 to 2034.

**Results:**

Globally, the number of DALYs due to cataract increased from 3,492,604 (95% uncertainty interval [UI] 2,481,846-4,719,629) in 1990 to 6,676,281 (95% UI 4,761,210-9,006,193) in 2019. The ASDRs due to cataract decreased from 93.17 (95% UI 66.14-125.32) in 1990 to 82.94 (95% UI 59.06-111.75) in 2019, with an average annual percentage change of –0.37 (95% CI –0.44 to –0.3; *P*<.001). Age, female sex, air pollution, smoking, high fasting plasma glucose levels, and a high body mass index were risk factors for the burden of cataracts. SDI and HDI were negatively correlated with ASDRs of cataracts, while PM_2.5_ and UVR were positively associated with them. Higher DALY rates were also associated with lower SDI (*R*^2^=0.1939; *P*<.001), lower HDI (*R*^2^=0.2828; *P*<.001), national PM_2.5_ concentration (*R*^2^=0.1874; *P*<.001), and ambient UVR levels (*R*^2^=0.2354; *P*<.001). The prediction model suggested that the number of DALYs due to cataract will continue to rise globally, while the cataract DALY rate will continue to decrease.

**Conclusions:**

While the ASDR of cataracts has decreased, there has been a notable increase in the number of DALYs over the past 30 years. Projections suggest that the global burden of cataracts will continue to rise over the next 15 years. To address this challenge, appropriate prevention and treatment policies must be implemented.

## Introduction

With the progressive aging of the world’s population, health issues, such as ocular health, have drawn more attention [1]. Cataracts are among the main causes of blindness globally, accounting for nearly half of all cases of blindness in low-income countries and 5% in high-income countries [2,3]. Recently, researchers have focused on the disability-adjusted life years (DALYs), which are defined as the sum of years lived with disability and years of life lost due to premature death and reflect the difference between the real state of health and the standard condition [4,5]. Moreover, DALYs attributed to cataracts exceed 90% in low-income countries and generally indicate the sum of years lived with disability, underscoring the significant impairments cataracts deal to the patients’ quality of life [4]. As the global burden of cataracts continues to grow due to the rise in global population and aging, this phenomenon will inevitably increase the economic burden, decrease the quality of life, and lead to cognitive function impairments [3,5]. Therefore, investigating the trends in the global cataract burden and associated risk factors will allow more targeted prevention strategies to be initiated, thereby promoting targeted prevention and treatment of cataracts.

A trend analysis of the Global Burden of Disease (GBD) Study 2019 data showed that the global burden of visual impairment due to cataracts exhibited a substantial increase, with the prevalence rate rising by 58.45% and the DALY rate increasing by 32.18% over the past 30 years [6]. Cataract is a multifactorial disease manifested by transparency loss of the eye lens, owing to tissue degradation and protein clumping, as well as pathogenic environmental and genetic factors [7]. Previous publications have reported risk factors such as aging, female sex, diabetes, excess weight, diet and nutritional status, smoking, drinking, exposure to ultraviolet light, socioeconomic status, and air pollution [8-23]. In addition, cataracts can also be innate, owing to a variety of genetic and molecular mechanisms [7]. However, the extent to which these risk factors affect cataracts remains unclear. Additionally, the long-term global trend of DALYs due to cataracts has not been visually demonstrated and analyzed, necessitating further research.

To address these gaps, we conducted an evaluation of the global trend in the DALYs of cataract by year, age, and sex from 1990 to 2019 and estimated its prediction over the next 15 years. Additionally, we conducted correlation analyses between age-standardized DALY rates (ASDRs) and the socio-demographic index (SDI) and human development index (HDI) separately. We then estimated the proportion of DALYs that could be attributed to several important risk factors for cataracts, including air pollution, smoking, high fasting plasma glucose, and a high BMI. Finally, the association between HDI, particulate matter <2.5 μm in diameter (PM_2.5_), and ultraviolet radiation (UVR) and ASDRs of cataracts was analyzed. Our results represented a significant extension of previous reports and offered valuable insights for future initiatives aimed at preventing cataracts.

## Methods

### Data Sources

This study was based on the GBD Study 2019, the United Nations Development Programme, and the WHO (World Health Organization) Global Health Observatory data repository. We collected related data on cataracts and SDI from the GBD database. The HDI data were obtained from the United Nations Development Programme, and concentrations of PM_2.5_ and average daily UVR data were collected from the WHO Global Health Observatory data repository. We estimated the global population using data from the GBD database.

### Study Variable Description

Study results are presented by number of DALYs, ASDRs, crude DALY rates, and average annual percentage changes (AAPCs) between 1990 and 2019 using 95% uncertainty intervals (UIs) and 95% CIs, age, sex, SDI, HDI, PM_2.5_ concentration, and UVR. The SDI gauges a nation’s economic development using per capita income distribution, average schooling years, and the fertility rate of women under 25 years of age [24]. The SDI ranges from 0 to 1, with higher values indicating greater economic advancement. The HDI was used as a measure of health, education, and living conditions [25]; it is calculated from life expectancy, education years, schooling expectancy, and per capita gross national income. HDI values range from 0 to 1, with higher values indicating greater socioeconomic development. The UNDP classifies countries into subgroups based on HDI: low (<0.550), moderate (0.550-0.699), high (0.700-0.799), and very high (≥0.800). The PM_2.5_ concentration is a common measurement for air pollution in a country and is defined as the annual average concentration of suspended fine particulate matter smaller than 2.5 μm in diameter. The UVR was defined as the average daily level of ambient solar UVR (in J/m^2^).

### Statistical Analysis

Temporal trends in DALYs due to cataract were assessed using the Joinpoint regression software (version 4.9.0.0; National Cancer Institute). This model uses segmented regression to capture disease distribution patterns over time. For each juncture point, the annual percentage change (APC) was computed, accompanied by a 95% CI. Additionally, the AAPC was determined as the weighted mean of individual APCs, offering a consolidated overview of the study period’s overall trend. In summary, a rising cataract rate was noted if both the APC and the lower 95% CI bound exceeded 0, while a declining rate was indicated if both values fell below 0. Linear regression analysis was used to determine the relationship between ASDR and SDI, HDI, national PM_2.5_, and ambient UVR. To predict DALYs due to cataract from 2020 to 2035, we used the Nordpred age-period-cohort model implemented in the Nordpred package in R (version 4.2.2; R Foundation for Statistical Computing). To validate the stability of the prediction results, the Bayesian age-period-cohort model was further applied to perform a sensitivity analysis using the R package “BAPC.” All statistical analyses and data visualization were conducted using R, the Joinpoint Regression Program, and GraphPad Prism 9.0 (GraphPad Software, Inc). A significance level of α=.05 was used, with *P*<.05 indicating statistical significance.

### Ethical Considerations

The authors received an exemption from ethical review from the institutional review board of the First Affiliated Hospital of Soochow University, and the study was also exempt from ethical review by the National Health Research Ethics Council of China. The research adhered to the principles of the Declaration of Helsinki. Due to the retrospective nature of the study, the requirement for informed consent regarding data processing and disease models was waived.

## Results

### Temporal Trends of Disease Burden Due to Cataract From 1990 to 2019

**Figure 1:**
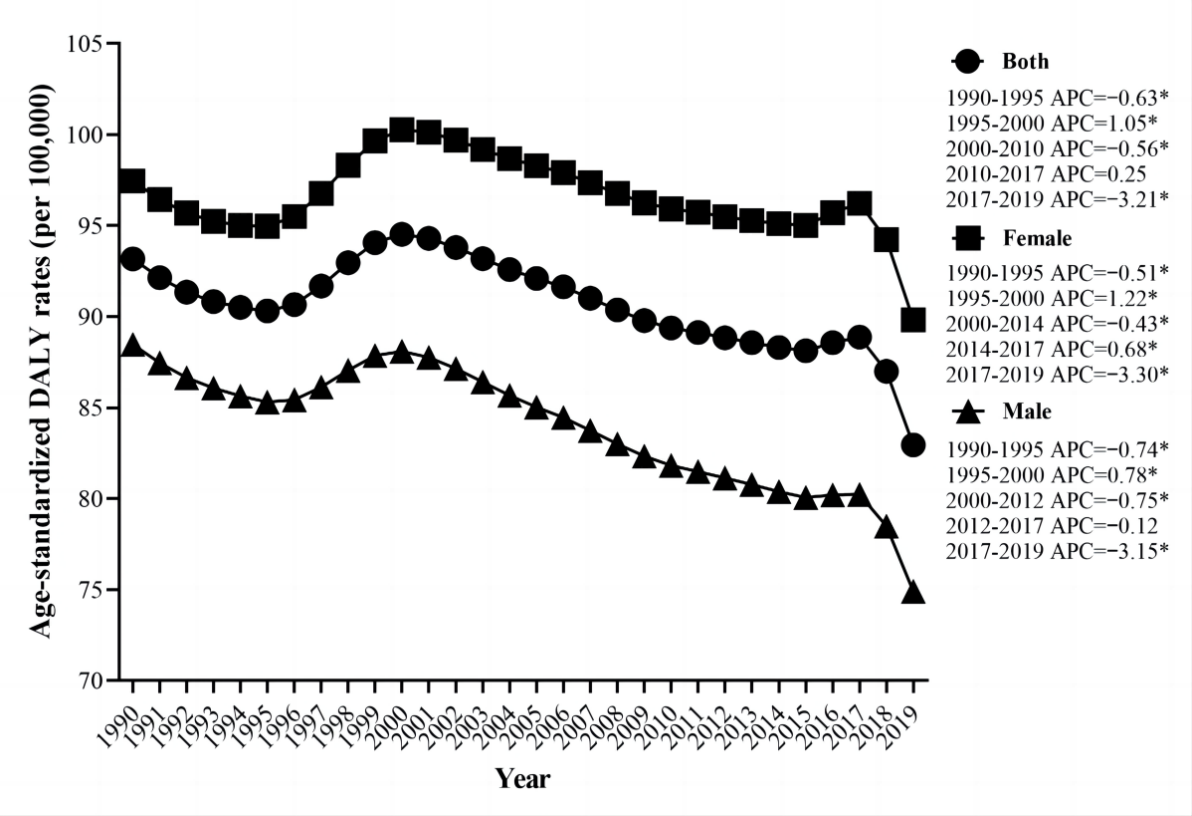
Joinpoint regression analysis of global age-standardized disability-adjusted life-years (DALYs) rates due to cataract for both female and male from 1990 to 2019. *P<.05; APC: annual percentage change.

Globally, the number of DALYs due to cataracts increased from 3,492,604 (95% UI 2,481,84-4,719,629) to 6,676,281.11 (95% UI 4,761,210-9,006,193) in 2019 (Table 1). The ASDR exhibited a dramatic decrease from 1990 to 1995 (APC=–0.63; *P*<.05), a dramatic increase from 1995 to 2000 (APC=1.05; *P*<.05), and another dramatic decrease from 2000 to 2010 (APC=–0.56; *P*<.05), followed by a slight increase from 2010 to 2017 (APC=0.25; *P*>.05). However, a remarkable decrease in ASDR was observed between 2017 and 2019 (APC=–3.21; *P*<.05) (Figure 1).

**Table 1:** Disability-adjusted life years (DALYs) of cataract and its average annual percentage changes from 1990 to 2019 at the global and regional levels.

Characteristics		1990		2019		1990-2019	*P* value
		Value (95% UI^a^)	ASDR^b^ (95% UI)	Number (95% UI)	ASDR (95% UI)	AAPC^c^ (95% CI)	
Global		3,492,605 (2,481,846-4,719,629)	93.17 (66.14-125.32)	6,676,281 (4,761,211-9,006,194)	82.94 (59.06-111.75)	–0.37 (–0.44 to –0.3)	<.001
**Sex**							
	Female	2,025,405 (1,442,842-2,729,748)	97.44 (69.34-130.79)	3,928,327 (2,798,531-5,275,330)	89.82 (63.97-120.6)	–0.25 (–0.33 to –0.16)	<.001
	Male	1,467,199 (1,038,553-1,989,939)	88.46 (62.56-118.7)	2,747,954 (1,954,020-3,725,738)	74.91 (53.35-101.18)	–0.54 (–0.6 to –0.49)	<.001
**SDI^d^ region**							
	High SDI	226,706 (161,075-309,553)	21.86 (15.5-29.71)	407,175 (289,682-556,589)	20.57 (14.49-28.1)	–0.2 (–0.23 to –0.17)	<.001
	High-middle SDI	527,854 (374,258-709,159)	54.05 (38.26-71.81)	1,079,895 (770,486-1,455,305)	53.72 (38.45-72.57)	0.03 (–0.1 to 0.15)	.687
	Middle SDI	1,238,750 (878,931-1,687,529)	139.91 (99.06-188.58)	2,469,946 (1,747,303-3,330,111)	107.61 (75.96-144.28)	–0.87 (–0.95 to –0.79)	<.001
	Low-middle SDI	1,126,634 (800,056-1,538,168)	216.76 (153.53-292.32)	2,009,356 (1,432,490-2,712,777)	160.43 (114.24-215.23)	–1 (–1.04 to –0.97)	<.001
	Low SDI	370,745 (264,016-506,463)	180.68 (128.63-244.99)	706,519 (502,216-953,371)	153.38 (108.77-206.59)	–0.55 (–0.57 to –0.54)	<.001
**South–East Asia, East Asia, and Oceania**							
	East Asia	453,089 (319,903-623,402)	63.9 (45-86.66)	1,097,095 (767,203-1,501,496)	57.37 (40.44-77.8)	–0.26 (–0.56 to –0.03)	.083
	Southeast Asia	632,885 (447,942-866,995)	279.06 (198.51-378.15)	1,079,918 (764,186-1,467,494)	195.67 (138.4-262.38)	–1.22 (–1.24 to –1.2)	<.001
	Oceania	4572 (3228-6213)	181.65 (128.83-242.25)	9854 (7084-13,440)	163.72 (116.76-222.47)	–0.38 (–0.45 to –0.31)	<.001
**Sub-Saharan Africa**							
	Eastern Sub-Saharan Africa	110,011 (78,056-152,266)	156.81 (112.06-215.18)	195,926 (138,353-268,562)	129.15 (91.78-176.19)	–0.67 (–0.7 to –0.63)	<.001
	Central Sub-Saharan Africa	5896 (4127-8269)	32.86 (22.99-45.35)	11,403 (7827-15,764)	26.13 (18.05-35.82)	–0.79 (–0.81 to –0.76)	<.001
	Southern Sub-Saharan Africa	30,885 (21,678-42,767)	119.28 (84.16-164.15)	41,255 (29,230-56,630)	80.48 (57.65-110.15)	–1.35 (–1.38 to –1.33)	<.001
	Western Sub-Saharan Africa	129,494 (91,237-176,192)	165.62 (117.94-224.66)	258,586 (182,989-352,960)	152.14 (107.71-205.16)	–0.3 (–0.34 to –0.26)	<.001
South Asia		1,303,763 (924,771-1,774,799)	273.27 (194.6-366.72)	2,544,708 (1,812,812-3,422,950)	198.39 (142.54-264.15)	–1.07 (–1.09 to –1.04)	<.001
**Latin America and Caribbean**							
	Caribbean	13,961 (9858-19,244)	56.3 (39.63-76.84)	22,146 (15,531-30,169)	42.9 (30.02-58.37)	–0.93 (–0.94 to –0.93)	<.001
	Central Latin America	67,300 (47,685-90,784)	90.64 (64.33-121.65)	146,616 (104,295-197,476)	64.7 (46-87.16)	–1.17 (–1.2 to –1.14)	<.001
	Tropical Latin America	76,104 (54,205-104,197)	96.82 (68.48-130.47)	164,285 (117,096-218,938)	70.86 (50.45-94.07)	–1.06 (–1.13 to –0.99)	<.001
	Andean Latin America	26,053 (18,322-35,146)	140.25 (99-188.37)	52,092 (36,890-69,969)	96.22 (68.28-129.43)	–1.31 (–1.45 to –1.17)	<.001
North Africa and Middle East		211,075 (149,404-288,143)	143.15 (101.05-195.59)	376,380 (264,296-513,801)	98.21 (69.44-134.06)	–1.3 (–1.32 to –1.27)	<.001
**Central Europe, Eastern Europe, and Central Asia**							
	Central Asia	32,930 (23,072-44,833)	77.49 (54.76-104.16)	41,305 (28,608-56,756)	66.22 (45.77-89.99)	–0.54 (–0.57 to –0.51)	<.001
	Eastern Europe	86,076 (60,611-115,280)	33.49 (23.53-44.81)	100,478 (70,300-136,392)	28.94 (20.29-39.35)	–0.5 (–0.52 to –0.48)	<.001
	Central Europe	28,961 (19,939-39,952)	21.26 (14.81-28.96)	42,190 (29,051-58,232)	19.36 (13.34-26.63)	–0.32 (–0.33 to –0.31)	<.001
**High-income regions**							
	Southern Latin America	16,633 (11,565-22,343)	38.56 (26.81-51.96)	27,402 (19,214-37,237)	32.5 (22.74-44.1)	–0.58 (–0.61 to –0.55)	<.001
	Western Europe	153,209 (107,373-210,703)	26.68 (18.64-36.59)	246,102 (172,914-338,143)	25.31 (17.75-34.81)	–0.18 (–0.2 to –0.16)	<.001
	North America	65,177 (46,278-88,463)	18.06 (12.84-24.71)	112,884 (80,414-152,299)	17.43 (12.34-23.78)	–0.12 (–0.15 to –0.08)	<.001
	Australasia	4791 (3315-6506)	21.32 (14.83-29.03)	10,416 (7336-14,137)	20.3 (14.25-27.82)	–0.17 (–0.21 to –0.13)	<.001
	Asia Pacific	39,741 (28,017-54,026)	21.09 (14.83-28.65)	95,238 (67,107-130,641)	19.5 (13.59-26.89)	–0.27 (–0.29 to –0.25)	<.001

^a^UI: uncertainty interval.

^b^ASDR: age-standardized DALY rate.

^c^AAPC: average annual percent change.

^d^SDI: socio-demographic index.

### Regional Differences in Disease Burden Due to Cataract

At the regional level in 1990 and 2019, the number of DALYs due to cataracts was highest in South Asia and lowest in Oceania (Table 1). The ASDR was highest in Southeast Asia in 1990 and highest in South Asia in 2019. From 1990 to 2019, the AAPC of ASDR in Southern Sub-Saharan Africa was lowest at –1.35 (95% CI –1.38 to –1.33; *P*<.001), followed by Andean Latin America at –1.31 (95% CI –1.45 to –1.17; *P*<.001). In contrast, the AAPC of ASDR in North America was highest at –0.12 (95% CI –0.15 to –0.08; *P*<.001).

### Age-Specific and Gender-Specific Disease Burden Due to Cataract

**Figure 2:**
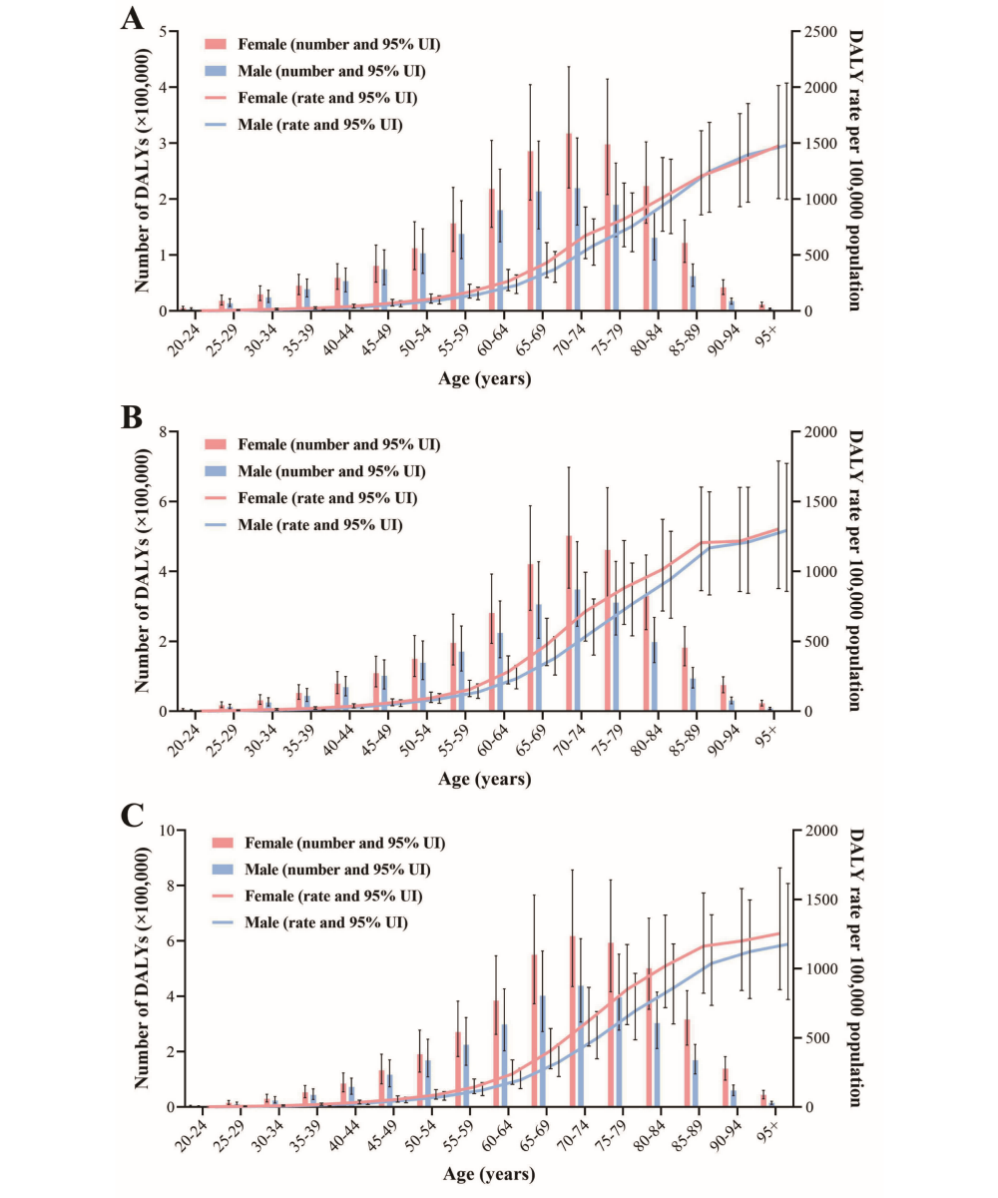
Global number of disability-adjusted life years (DALYs) and crude DALY rates due to cataract by age and sex in (A) 1990, (B) 2005, and (C) 2019. The black bar indicates the upper and lower limits of the 95% uncertainty intervals (UIs) for females and males, respectively.

With increasing age, the global number of DALYs due to cataracts gradually increased (up to the age of 75 years) in 1990, reaching a peak at the ages of 70-74 years, after which it declined with increasing age. Similar trends were observed in 2005 and 2019. The DALYs due to cataracts were relatively low in individuals aged less than 30 years or more than 95 years. The crude DALY rates of cataracts rose with increasing age, with the fastest increment observed between the ages of 60 and 85 years in 1990. In 2005 and 2019, the general trend of the crude DALY rates was similar to 1990; however, the rate of increase after the age of 85 years slowed down more significantly (Figure 2). The global number of DALYs due to cataracts was lower in males than in females in 1990 and 2019, while ASDR was also smaller in males (Table 1). Moreover, the ASDR of male cataracts decreased more than that of females between 1990 and 2019, with AAPCs of –0.54 (95% CI –0.60 to –0.49; *P*<.001) and –0.25 (95% CI –0.33 to –0.16; *P*<.001), respectively (Table 1 and Figure 1).

### Characteristics of the Cataract Burden Attributable to Risk Factors

**Figure 3:**
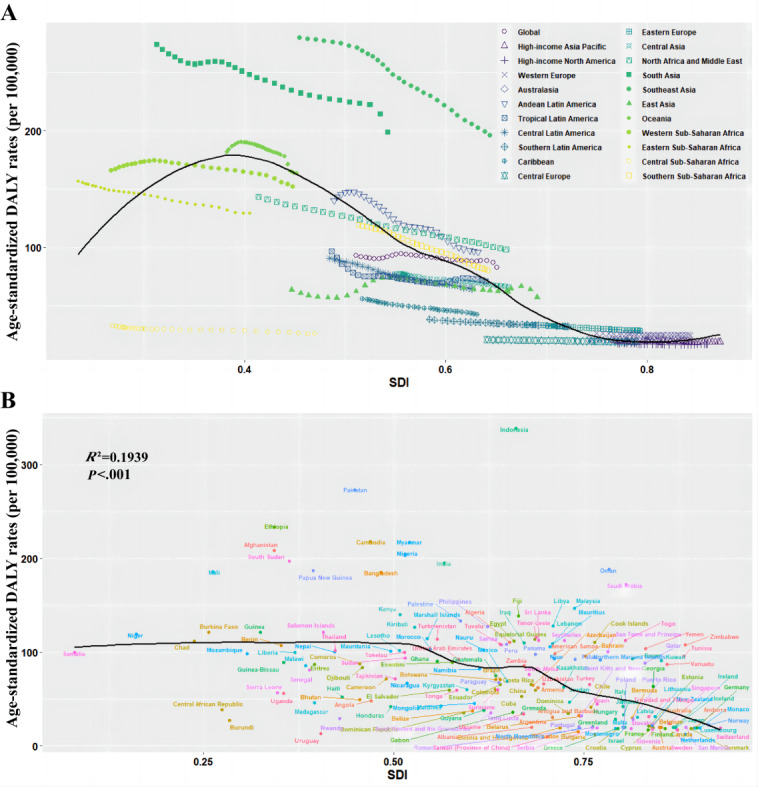
Age-standardized disability-adjusted life year (DALY) rate (per 100,000 persons) of cataract by socio-demographic index (SDI) for (A) 21 Global Burden of Disease (GBD) Study regions from 1990 to 2019, and (B) 204 countries and territories in 2019. The black line represents the expected values based on age-standardized DALY rates and SDI in all locations.

**Figure 4:**
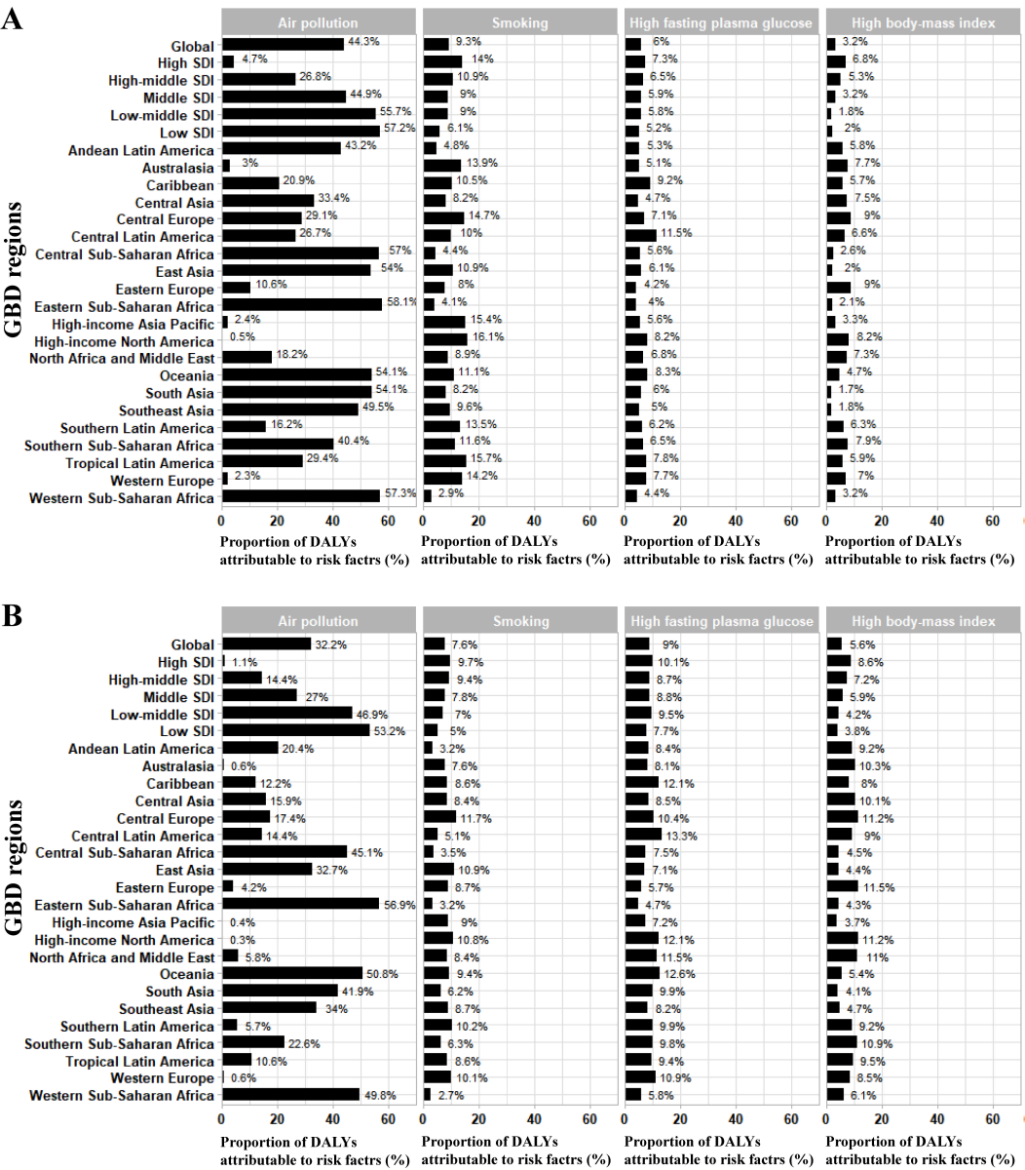
The proportion of cataract disability-adjusted life year (DALY) attributable to air pollution, smoking, high fasting plasma glucose, and high body-mass index for (A) 21 Global Burden of Disease (GBD) Study regions in 1990 and (B) 2019. SDI: socio-demographic index.

Areas with high SDI have lower ASDRs for cataracts than those with low SDI, with high-income North America having the lowest ASDRs and South Asia having the highest between 1990 and 2019 (Table 1 and Figure 3A). In addition, the ASDRs of cataracts were lower in countries with a high SDI than in those with a low SDI. Linear regression analysis revealed that ASDRs were negatively correlated with SDI (*R*^2^=0.1939; *P*<.001; Figure 3B). ASDRs of cataracts due to air pollution, smoking, high fasting plasma glucose, and high BMI in 21 GBD regions in 2019 were obtained and presented in Figure 4. Apart from high SDI regions, air pollution accounted for the largest proportion of the 4 listed factors leading to cataracts, which increased as the SDI declined. The scatter plots between ASDRs of cataract and other country-level indicators were presented in Multimedia Appendix 1, and a linear trend was observed between ASDRs of cataract and HDI level (*R*^2^=0.2828; *P*<.001), national PM_2.5_ concentration (*R*^2^=0.1874; *P*<.001), and ambient UVR (*R*^2^=0.2354; *P*<.001). Based on the aforementioned results, HDI, national PM_2.5_ concentration, and ambient UVR were included as independent variables to construct the multivariate linear regression model for ASDRs. A significant relationship was identified (*P*<.001), and this model accounted for 37.6% of the variance in the national differences in cataract burden.

### Predictions of DALYs Due to Cataract

**Figure 5:**
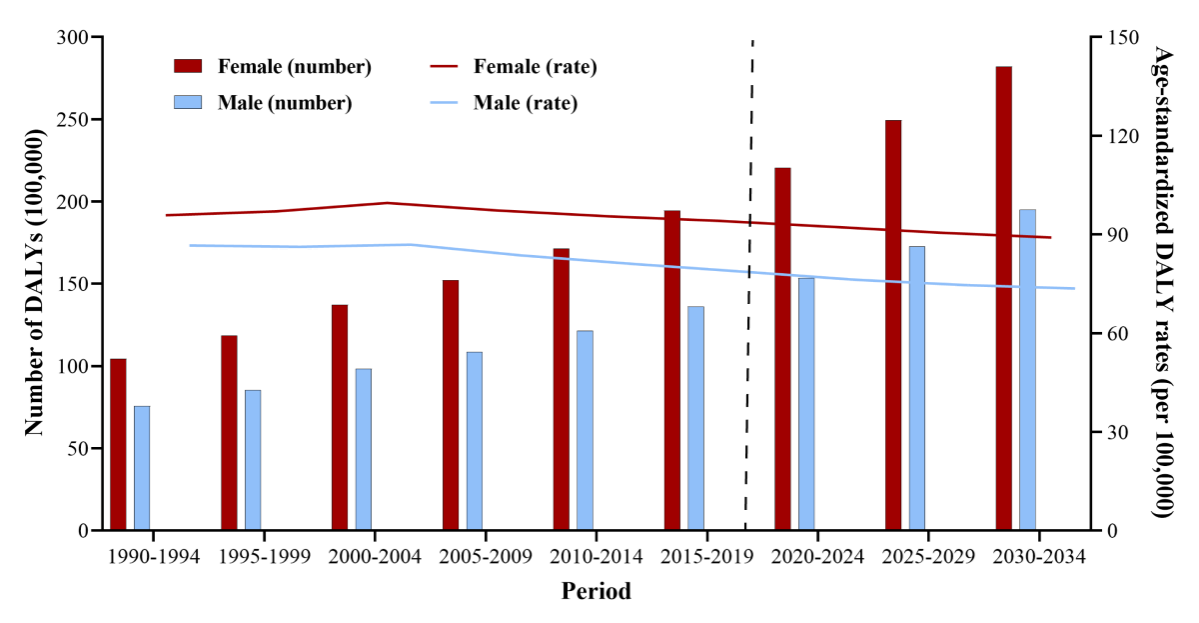
Trends in global number of disability-adjusted life years (DALYs) and age-standardized DALY rates (per 100,000 persons) due to cataract by sex: observed (1990-2019) and predicted (2020-2034).

We predicted that, due to population growth and aging, the DALYs due to cataracts for both sexes are expected to continue to increase over the next 15 years. Further, over the next 15 years, a decrease in the ASDRs of cataracts is expected, particularly among women (Figure 5 and Multimedia Appendix 2). While the patterns of changes in DALY rates in men were similar to those in women, the DALY rates of cataracts were notably lower among men. To assess the robustness of our predictions, we further performed a sensitivity analysis using the BAPC package to predict the future global burden of cataracts; the results showed consistent trends with the abovementioned results (Multimedia Appendix 3).

## Discussion

### Principal Findings

This study reported on the number of DALYs and ASDRs due to cataracts from 1990 to 2019, revealing disparities by region, nation, age, and sex. Although the ASDRs decreased overall, the overall DALYs due to cataracts still increased. Our findings showed that over the last 3 decades, higher DALY rates were also associated with lower SDI, HDI, national PM_2.5_, and ambient UVR. Furthermore, we predicted that the global burden of cataracts will continue to increase over the next 15 years due to the growth and aging of the global population. To the best of our knowledge, this report acts as an essential complement to previous studies, and the results of our research can provide crucial insights into evidence-based health care planning and reduce the global burden of cataracts by allocating resources to cataract prevention.

Global policy making and economic restructuring can guide the prevention and treatment of cataract. Initially, the WHO and International Agency for the Prevention of Blindness started the VISION 2020 global initiative in 1999 to prevent, control, and eliminate avoidable blindness at the national level by 2020 [26]. In line with earlier studies, the number of DALYs in cataracts has increased globally over the last 30 years, although the ASDRs fell between 1990 and 2019 [6,10,20]. According to our findings, low-income regions exhibited higher ASDRs compared to high-income regions, indicating a greater burden of cataract in the former. Several factors could potentially explain this pattern, including elevated UVR levels in southern countries and the prevalence of conditions such as arterial hypertension and diabetes, which might contribute to the proliferation of this ailment. Notably, in response to VISION 2020, high-income regions allocated funding for diverse programs aimed at reducing rates of low vision and blindness. The adoption of more proactive diagnostics, early detection, and treatment strategies could potentially bolster the performance of high-income regions [27]. Addressing this phenomenon warrants increased attention and further investigations into the mechanisms underlying these disparities.

Although previous studies have implicated many risk factors for cataracts, age is uniquely important. Cataracts can be divided by age-related, pediatric, and secondary causes. Age-related cataract is the most common type in adults, starting between the ages of 45 and 50 years [28]. Aging is the most meaningful risk factor for cataract because structural and functional changes to the lens can promote cataract development. The lens consists of soluble proteins, and the fiber cell organization in the lens is tight, which enables intercellular space reduction. These mechanisms prevent light content and support lens transparency. Increasing age leads to slower protein metabolism, reducing their solubilization, which in turn impairs lens transparency and may cause other pathological risk factors to have cumulatively detrimental effects [29,30].

According to our findings, both the number of DALYs and ASDRs caused by cataracts in females was greater than that in males from 1990 to 2019. The following is consistent with other studies that found similar situations of sex disparity in ocular diseases, such as cataracts, age-related macular degeneration, and diabetic retinopathy [5,21,30,31]. Possible explanations for these results include differences in follow-up time points and longer life expectancies in female patients [10]. Another explanation could be related to the inequality between men and women, as women can be disadvantaged in terms of access to education, employment opportunities, income distribution, and health care services [32]. Other research has also focused on the function of estrogen in cataract formation, which has been shown to play a role due to antioxidant effects on reactive oxygen species, which are important factors for cataracts. Menopause is hypothesized to cause an elevated risk of cataract in older females, caused by the withdrawal effect [9]. More attention and further studies regarding female eye care are required to explore the possible mechanisms between sex and cataract.

Furthermore, health inequalities are largely caused by socioeconomic disparities. According to our findings, the ASDRs of cataracts varied significantly between regions and nations and were lower in high-income regions and countries, while SDI and HDI were negatively correlated with cataract ASDR. A higher quality of eye care and increasing opportunities for cataract surgery may explain this association in countries with high SDI and HDI scores. Although surgery is the definitive therapy for cataract, many major barriers to cataract treatment still exist [33]. One aspect is that the outcome of cataract surgery is related to the quality of the surgery. Access to quality cataract surgery remains difficult in many countries, while the coverage of cataract surgery is influenced by GDP and health expenditure [18,20]. The lack of education and medical knowledge, as well as the cost of surgery, may discourage patients in low-income areas from choosing treatment [19]. As a result, the cataract surgical rate is over 10,000 in developed countries and below 500 in low-income countries [34]. Thus, in low-income countries, the emphasis on reducing the burden of cataracts in the formation of health policies should be as important as in high-income countries.

We also found that air pollution, smoking, high fasting plasma glucose, and high BMI were all significant cataract risk factors that contributed to most of the burden. In this study, air pollution referred mainly to household air pollution from solid fuels, which was associated with a higher risk of cataracts in agreement with previous studies [35,36]. Smoking is a risk factor for many diseases, including cataracts, glaucoma, and age-related macular degeneration*,* with studies reporting an elevated risk of nuclear sclerotic cataract in patients of both sexes [37-39]. Tobacco contains large quantities of toxic heavy metals, including cadmium, lead, and copper, and the accumulation of these metals on the lens can eventually contribute to cataract development [15,40,41]. More specifically, the ratio of the indirect impact of tobacco to the total impact of cadmium exceeded 50% [42]. This study also indicated that high fasting blood glucose levels were correlated with cataracts. The Blue Mountains Eye Study recently demonstrated that fasting blood glucose levels were associated with long-term cortical cataract formation and long-term progression of cortical, nuclear, and posterior subcapsular cataracts, which is consistent with our findings [43]. Similarly, the Beaver Dam Eye Study evaluated diabetic participants as having an increased risk of developing cataracts [44]. The study proposed strategies to address the increased risk of cataracts in diabetic patients, which include targeting the polyol pathway, oxidative stress, and nonenzymatic glycation of lens proteins [45,46]. Moreover, a study indicated that diabetic retinopathy is an independent risk factor for cataract formation in patients with type 2 diabetes, alongside BMI, HbA_1c_, and insulin usage [12]. The Blue Mountain Eye Study also showed an increased risk of cataract in relation to metabolic syndrome [47]. Therefore, obesity is correlated with increased DALY rates in cataract. Obesity, especially central obesity, is associated with insulin resistance, which indirectly leads to type 2 diabetes [48]. In addition, the lipid composition of the lens changes during cataract formation, possibly because high BMI and excess body weight alter lipid metabolism in the lens [14]. The results of this study may have implications for public health, as cataracts are susceptible to air pollution, smoking, high fasting plasma glucose, and high BMI, and increased health awareness may be a cost-effective intervention to prevent blindness in patients with cataract.

This study illustrated that exposure to PM_2.5_ and solar UVR accelerated cataract development. Current evidence suggests that air pollution may affect ocular diseases, including cataracts, age-related macular degeneration, and glaucoma [22]. After exposure to air pollutants such as PM_2.5_, NO_2_ (nitrogen dioxide), and NO_x_ (nitrogen oxide), the risk of cataract surgery increased by 5%, but the effects were relatively small [49]. The most common assessment of air pollution is PM_2.5_. The Canadian Longitudinal Study on Aging illustrated that PM_2.5_ had a borderline-adjusted correlation with cataracts [23]. Evidence suggests that exposure to high-dose UVR can cause acute photokeratitis and photoconjunctivitis while chronic exposure to low-dose UVR can cause cataract, pterygium, and corneal and conjunctival squamous cell carcinoma [50]*.* Interestingly, cataract DALY rates were lower in urbanized areas because the work of most individuals could be completed indoors, and their UVR exposure could be at a lower level [51]. Evidence suggests that oxidation accumulation, antioxidant potential, and repair function of the lens decrease with age, which are all related to age-related cataracts. The most significant cause of cataract may be the formation of reactive oxygen species caused by UVR. This decreased the activity of a series of enzymes that catalyze the reduction of hydrogen peroxide and peroxide radicals [50]. Our results indicate that improving the living environment and increasing UV radiation protection may be effective interventions to delay the occurrence of cataract blindness, while the pathogenic roles of PM_2.5_ and UV light in cataracts require further study.

While the DALY rates for cataract decreased overall in most age and sex groups between 1990 and 2019, there was a significant increase in the total number of DALYs due to cataract. This upward trend in cataract DALYs, coupled with an aging population and expanding economy, is likely to lead to a greater economic burden on health care. We anticipate that the number of DALYs due to cataract will continue to rise steadily from 2020 to 2034. In light of the anticipated increase in the global burden of cataract, efforts should be made to strengthen prevention and management strategies for both established and emerging risk factors such as air pollution, smoking, and UV radiation. Effective global policies aimed at reducing the prevalence of these risk factors could play a critical role in mitigating the impact of cataract and reduce its burden.

### Study Limitations

Although this study enabled us to examine the global cataract burden and its relationship to risk factors with high confidence, some limitations should be noted. First, all data come from the GBD 2019, UNDP, and GHO. As such, the authenticity and reliability of the results depend on the data source. Second, cataract is a multifactorial disease, and some key factors, such as congenital, traumatic, and drug-induced factors, have not been analyzed in this study; therefore, the factors included in our model may also be affected. Third, the prevalence and incidence of cataracts were not investigated in this study. Moreover, this study did not conduct a decomposition analysis of the disease burden of cataract over time. This omission indicates a potential gap in understanding the complex dynamics underlying the changing disease burden of cataract. Therefore, it is essential to explore and gather more information on cataract to improve our understanding of this important public health issue.

### Conclusions

Over the past 3 decades, the global burden of cataract has increased, as evidenced by the increasing number of DALYs. Furthermore, given the changing demographics of the world, this trend is expected to persist over the coming 15 years. Age, female sex, air pollution, smoking, high fasting plasma glucose, high BMI, UVR, and low socioeconomic status were risk factors for the burden of cataracts. Our findings showed that the older adults and female populations in low-income countries have a greater burden caused by cataract; therefore, health services for cataract should be strengthened for these populations. Effective resource allocation and health-service planning will be essential in managing the anticipated increase in the global burden of cataract in the next 15 years.

## Appendix

Multimedia Appendix 1 Associations between age-standardized disability-adjusted life-year (DALY) rates (per 100,000 persons) of cataract and HDI (A), national PM2.5 (B), and ambient ultraviolet radiation (C) in 2019. HDI: human development index; PM2.5: particle matter <2.5 μm in diameter.

Multimedia Appendix 2 Trends in global number of disability-adjusted life-years (DALYs) and age-standardized DALY rates (per 100,000 persons) due to cataract by sex: observed (1990-2019) and predicted (2020-2034).

Multimedia Appendix 3 Trends in global number of disability-adjusted life-years (DALYs) and age-standardized DALY rates (per 100,000 persons) due to cataract by sex: observed (1990-2019) and predicted (2020-2034).

## Abbreviations

AAPC: average annual percentage change
APC: annual percentage change
ASDR: age-standardized DALY rate
DALY: disability-adjusted life year
GBD: Global Burden of Disease
HDI: human development index
NO2: nitrogen dioxide
NOx: nitrogen oxide
PM2.5: particulate matter <2.5 μm in diameter
SDI: socio-demographic index
UVR: ultraviolet radiation
UI: uncertainty interval
WHO: World Health Organization

## Data Availability

The data analyzed during this study are available from the corresponding author on reasonable request.
