# Supplementary figures and images for: Changing Trends in the Global Burden of Cataract Over the Past 30 Years: Retrospective Data Analysis of the Global Burden of Disease Study 2019
Source: JMIR Public Health Surveill. 2023 Dec 5;9:e47349. doi: 10.2196/47349 (PMC10731550; doi:10.2196/47349)

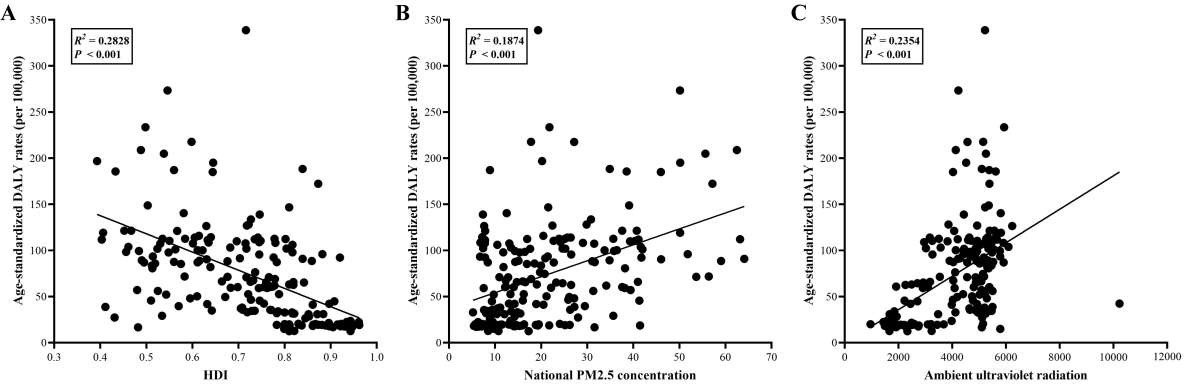

Supplement: Multimedia Appendix 1 [file publichealth_v9i1e47349_app1.png]

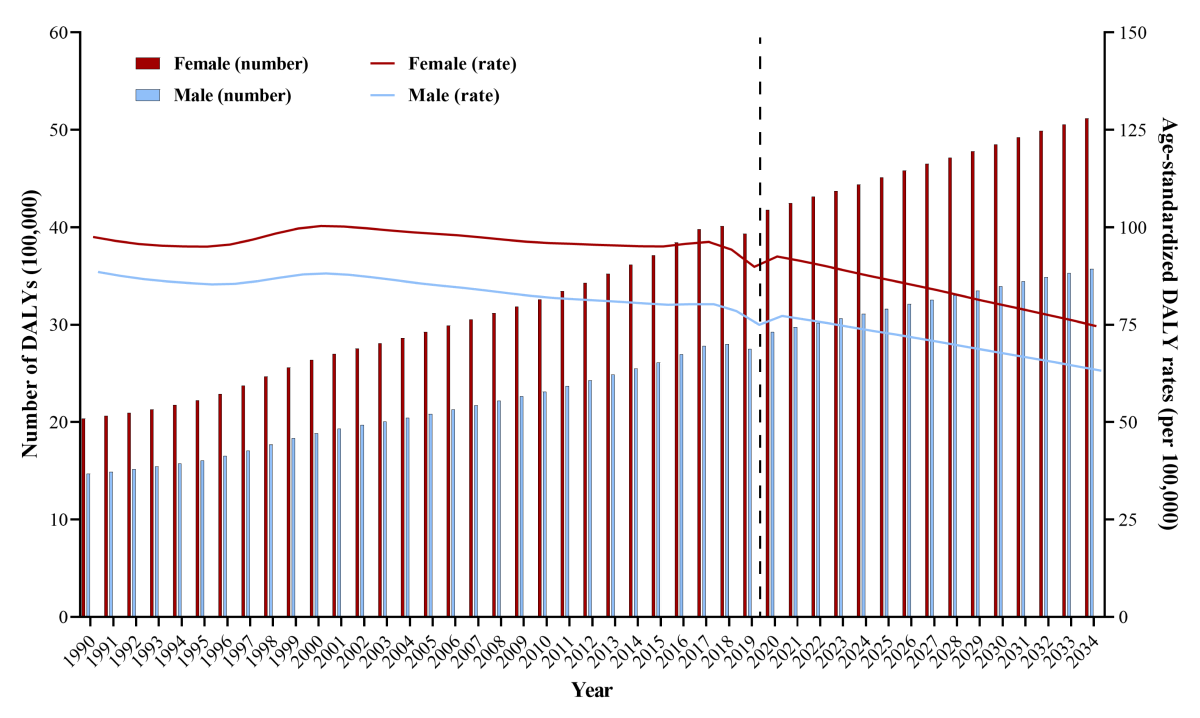

Supplement: Multimedia Appendix 3 [file publichealth_v9i1e47349_app3.png]
