# Supplementary material for: Changing Trends in the Global Burden of Cataract Over the Past 30 Years: Retrospective Data Analysis of the Global Burden of Disease Study 2019
Source: JMIR Public Health Surveill. 2023 Dec 5;9:e47349. doi: 10.2196/47349 (PMC10731550; doi:10.2196/47349)
Supplement: Multimedia Appendix 2 [file publichealth_v9i1e47349_app2.doc]

Table S1. Trends in global number of disability-adjusted life-years and age-standardized disability-adjusted life-year rates (per 100,000 persons) due to cataract by sex: observed (1990-2019) and predicted (2020-2034)

| **Periods** | **Female** | | **Male** | |
| --- | --- | --- | --- | --- |
|  | **Number** | **ASDR^a^** | **Number** | **ASDR** |
| 1990-1994 | 10447048 | 96 | 7581886 | 87 |
| 1995-1999 | 11865236 | 97 | 8561044 | 86 |
| 2000-2004 | 13716988 | 100 | 9826642 | 87 |
| 2005-2009 | 15232772 | 97 | 10861367 | 84 |
| 2010-2014 | 17136810 | 96 | 12140753 | 81 |
| 2015-2019 | 19453709 | 94 | 13628885 | 79 |
| 2020-2024 | 22052220 | 92 | 15343567 | 76 |
| 2025-2029 | 24935817 | 91 | 17273919 | 75 |
| 2030-2034 | 28207729 | 89 | 19501432 | 74 |

^a^ASDR: age-standardized DALY rate.
